# Direct and Indirect Effect of TGFβ on Treg Transendothelial Recruitment in HCC Tissue Microenvironment

**DOI:** 10.3390/ijms222111765

**Published:** 2021-10-29

**Authors:** Francesco Dituri, Serena Mancarella, Grazia Serino, Nada Chaoul, Luigi Giovanni Lupo, Erica Villa, Isabel Fabregat, Gianluigi Giannelli

**Affiliations:** 1 National Institute for Gastroenterology, IRCCS “S. De Bellis” Research Hospital, 70013 Castellana Grotte, Italy; serena.mancarella@irccsdebellis.it (S.M.); grazia.serino@irccsdebellis.it (G.S.); gianluigi.giannelli@irccsdebellis.it (G.G.); 2 Department of Emergency and Organ Transplant, School and Chair of Allergology and Clinical Immunology, University of Bari Medical School, 70124 Bari, Italy; nada.chaoul@uniba.it; 3 Department of General Surgery and Liver Transplantation, University of Bari Medical School, Policlinico, Piazza Giulio Cesare 14, 70124 Bari, Italy; luigigiovanni.lupo@uniba.it; 4 Gastroenterology Unit, Department of Internal Medicine, University of Modena and Reggio Emilia, 41121 Modena, Italy; erica.villa@unimore.it; 5 Oncobell Program, Bellvitge Biomedical Research Institute (IDIBELL), CIBEREHD and University of Barcelona, 08908 L’Hospitalet de Llobregat, Spain; ifabregat@idibell.cat

**Keywords:** HCC, TGF beta, cancer immunity, regulatory T cells, cancer-associated fibroblasts, transendothelial migration, secretome

## Abstract

The balance between anti-tumor and tumor-promoting immune cells, such as CD4+ Th1 and regulatory T cells (Tregs), respectively, is assumed to dictate the progression of hepatocellular carcinoma (HCC). The transforming growth factor beta (TGFβ) markedly shapes the HCC microenvironment, regulating the activation state of multiple leukocyte subsets and driving the differentiation of cancer associated fibroblasts (CAFs). The fibrotic (desmoplastic) reaction in HCC tissue strongly depends on CAFs activity. In this study, we attempted to assess the role of TGFβ on transendothelial migration of Th1-oriented and Treg-oriented CD4+ T cells via a direct or indirect, CAF-mediated mechanisms, respectively. We found that the blockage of TGFβ receptor I-dependent signaling in Tregs resulted in impaired transendothelial migration (TEM) of these cells. Interestingly, the secretome of TGFβ-treated CAFs inhibited the TEM of Tregs but not Th1 cells, in comparison to the secretome of untreated CAFs. In addition, we found a significant inverse correlation between alpha-SMA and FoxP3 (marker of Tregs) mRNA expression in a microarray analysis involving 78 HCCs, thus suggesting that TGFβ-activated stromal cells may counteract the trafficking of Tregs into the tumor. The apparent dual behavior of TGFβ as both pro- and anti-tumorigenic cytokines may add a further level of complexity to the mechanisms that regulate the interactions among cancerous, stromal, and immune cells within HCC, as well as other solid tumors, and contribute to better manipulation of the TGFβ signaling as a therapeutic target in HCC patients.

## 1. Introduction

The micro-environment of HCC, like that of other solid tumors, is a complex milieu populated by diverse cell types, other than cancer cells, which interact with each other through different ways (direct cell–cell contact, secreted growth factors, cytokines, and chemokines) [1,2]. The stromal compartment of HCC is mainly represented by fibroblasts and hepatic stellate cells (HSCs), which respond to multiple stimuli by switching between different phenotypes and secreting soluble factors that target other cell types in the tumor. The of cancer-associated fibroblasts (CAFs) designation includes cells not ascribable to epithelial or immune cells, which display a mesenchymal-like morphology and secrete significant amounts of proteins of extracellular matrix, particularly upon stimulation by specific stimuli (growth factors, cytokines, and so on) [3]. TGFβ is a multifunctional cytokine that has gained enormous interest in the last decades, due to its pivotal involvement in regulating both physiological and pathological processes, such as organogenesis, immune tolerance, synthesis of mucosa-restricted immunoglobulins (IgA), wound healing, and cancer, although many aspects of its roles in these contexts have yet to be elucidated [4,5,6]. TGFβ shows a strong and widespread overexpression in fibrotic liver and HCC, in comparison to normal liver, where its presence is virtually undetectable [7,8]. Invasion of cancerous hepatocytes and angiogenesis are promoted or enhanced by TGFβ in HCC [9,10]. TGFβ can contribute to progression of HCC also by stimulating CAFs to proliferate and release ECM proteins (collagens, fibronectin, laminins) or cytokines/chemokines, including CXCL6 and TGFβ itself [11,12,13]. Noteworthy, in synergy with other cytokines, TGFβ drives the polarization of immune cells toward specific commitment pathways, some of which generate subsets of leukocytes that are reported to play prominent roles in cancer progression. Regulatory CD4+ CD25+ FoxP3+ T cells (commonly designated as Tregs) are a subtype of a differentiated array of regulatory CD4+ T cells, which are generated mainly in the thymus (natural, or nTregs) and secondary lymphoid organs, including spleen, lymph nodes, and Peyer patches (induced, or iTregs) [14,15,16]. These cells work to hinder unwanted immune reactions, such as autoimmunity, or quench inflammation, in order to avoid excessive tissue damage, once the triggers are removed [17]. However, a role of Tregs as supporters of progression of several solid cancers, including HCC, has been widely reported on the basis of their ability to suppress the anti-tumor arm of immune response [18,19]. TGFβ has long been recognized as a major driver in CD4 T cell engagement towards a Treg lineage [15,20,21]. The capacity of TGFβ to induce the polarization of intratumor Tregs in HCC is to some extent responsible for the tumor-supporting activity by this cytokine [22]. Although the functions of TGFβ in regulating phenotypical polarization and activation status of immune cells in HCC has been extensively investigated, the role of “TGFβ-educated” HCC stromal cells in the recruitment and homing of tumor supporting and limiting lymphocytes has not yet been explored. Here, we investigated the direct and indirect CAF-mediated effects of TGFβ on the motility across an endothelial layer of Th1 and Treg-polarized CD4 T cells, as they are reportedly involved in anti-tumor and tumor promoting processes in HCC, respectively. Transendothelial migration (TEM) is a crucial step that allows leukocytes to leave the bloodstream to home within inflamed/infected tissues as well as sites of tumor onset and development.

## 2. Results

### 2.1. Commitment of CD4 T Cells toward Th1/Treg Phenotype and Preparation of HCC CAF Secretome

**Figure 1:**
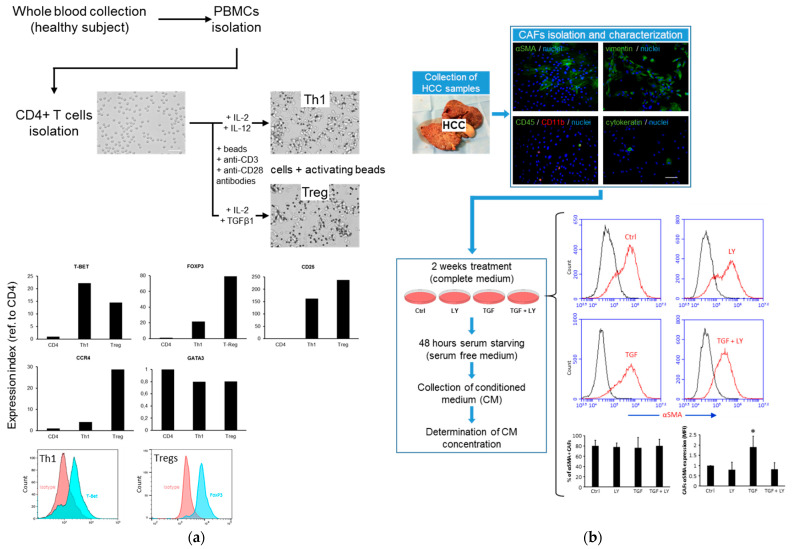
(**a**) Procedure for generating Th1- and Treg-oriented CD4 T cells. The expression of representative markers of Th1 (T-bet), Treg (FoxP3, CD25, CCD4), and Th2 (GATA3) commitment were analyzed by qPCR and flow cytometry. Expression data of qPCR were normalized on total RNA prior reverse transcription. (**b**) CAF isolation, characterization, and subsequent treatment for obtaining conditioned medium. Vimentin was visualized to confirm the fibroblast phenotype, and αSMA was evidenced to assess the partially myo-fibroblast differentiation. Only little contaminating immune cells (CD45+, CD11b+) or cholangiocytes (cytokeratin+) were evidenced. αSMA expression increased in CAFs following 2 weeks of TGFβ stimulation. Variation of percentage of αSMA+ cells and αSMA expression as a result of TGFβ stimulation were evaluated by flow cytometry analysis. Flow cytometry analysis of αSMA in CAFs was performed in cells from four HCC patients (MFI = mean fluorescence intensity). * = *p* < 0.05 (*t*-test, paired, two tailed).

Th1- and Treg-oriented CD4 T cells were generated, starting from CD4 cells isolated from peripheral blood mononuclear cells of a healthy subject, which were then stimulated with CD3/CD28-conjugated beads to simulate antigen-presenting cells in the presence of interleukin-2 (IL-2) and IL-12 for Th1 differentiation, or in the presence of IL-2 and TGFβ1 to obtain Tregs (Figure 1a). The analysis of expression of representative markers of Th1 (T-bet) and Treg (FoxP3, CD25, CCR4) by qPCR and flow cytometry confirmed the polarization of CD4 T cells toward these phenotypes. CAFs were isolated from freshly collected surgical HCC specimens and then characterized for the expression of typical related mesenchymal markers (vimentin, αSMA). These cells were incubated for 14 days in the presence/absence of TGFβ1 and the TGBβ receptor I inhibitor LY2157299 (LY), and then they were serum-starved and incubated for an additional 48 h to allow for secreted protein (secretome) enrichment in the conditional medium (CM). The chronical TGFβ1 treatment did not result in a further differentiation of αSMA- fibroblasts in αSMA+ myo-fibroblasts, which represented a yet major subset of the whole original CAF population. Rather, TGFβ1 stimulation further resulted in an increased αSMA expression level in αSMA+ CAFs, with this effect being reversed by LY2157299 (10 µM) (Figure 1b).

### 2.2. TGFβ1 Direct and Indirect (CAF-Mediated) Effects on Transendothelial Migration of Tregs

**Figure 2:**
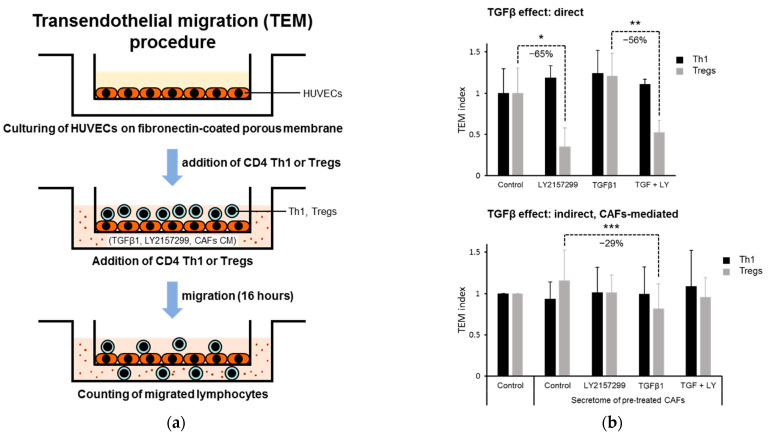
(**a**) Transendothelial migration (TEM) assay procedure. (**b**) TGFβ direct and indirect (CAF-mediated) effects on in vitro trensendothelial migration (TEM) of CD4+ Th1 and Tregs. (upper) Direct role of TGFβ on TEM of Th1 and Tregs. Active TGFβRI-dependent signaling was required for effective TEM of Tregs, but not Th1. LY21457299 (LY) significantly impaired the TEM of Tregs both in the absence or presence of TGFβ1. Exogenously added TGFβ1 did not significantly increase the TEM of Tregs over control, likely due to maximal activity of Tregs-derived TGFβ. The assay was conducted in quadruplicate. (lower) The conditioned medium (CM) from untreated CAFs (Control_CM), used as chemoattractant in the TEM assay, did not influence the TEM of both Th1 and Tregs in comparison with the serum-free medium. CAFs CM from CAFs previously treated with TGFβ1, or TGFβ1 + LY2157299 significantly inhibited the migration of Tregs alone, compared with Control_CM. Data are expressed as means ± SD of 8 independent TEM tests conducted using conditioned media of CAFs isolated from as many HCC patients. *t*-test (two tailed, unpaired for panel B, upper, or paired for panel B, lower): * *p* < 0.05; ** *p* < 0.01; *** *p* < 0.001.

Transendothelial migration (TEM) of Th1/Tregs-polarized CD4 T cells was simulated by performing a Transwell-based assay. Human umbilical vein endothelial cells (HUVECs) were used to mimic the microvascular endothelial layer (Figure 2a). The direct effect of TGFβ active/inactive pathway in Th1/Tregs-oriented CD4 T cells was analyzed. While Th1 did not appear to sense the presence of TGFβ or of the inhibitor of TGFβ receptor I LY2157299, the blockage of activation of this receptor by LY2157299 in Tregs markedly reduced their TEM both in the presence and absence of TGFβ (−65% and −56%, respectively), suggesting the essential role of activated TGFβ signaling in Tregs to perform TEM. The lack of effect upon TGFβ addition of the TEM of Tregs is likely due to sufficient Tregs self-production and autocrine feedback action of this cytokine by Tregs (Figure 2b, upper). We then analyzed the role of secretome from untreated or TGFβ1 and/or LY2157299 pre-treated CAF secretome on the TEM of Th1 and Tregs. Migration of both Th1- and Treg-oriented CD4+ T cells was not affected by CAFs_Control CM compared to serum-free medium, suggesting that the cytokines/chemokines secreted by the lymphocytes themselves were sufficient to induce maximum motility in this in vitro model (Figure 2b, lower). Moreover, CAFs_Control CM obtained by using filters with cut-off > 3 kDa did not increase the TEM compared to CAFs_Control CM cut-off > 10 kDa, demonstrating that partial loss of chemokines (which range from 8 to 10 kDa MW) during the filtering of CM does not result in the inability of T cells to migrate more in the presence of CM used as a chemoattractant than in SF medium (Figure S1). More importantly, CM from TGFβ1-treated CAFs significantly inhibited the TEM of Treg cells in comparison with control CM (−29%), while CM from LY2157299-treated CAFs was ineffective. By contrast, CM from CAFs subjected to TGFβ1 and/or LY2157299 treatment did not significantly change Th1 cell migration performance compared to CAFs_control CM. To evaluate the contribution of endothelial cells to the migration effects of CAFs_CM of Th1 and Tregs, we conducted a parallel in vitro migration assay in the absence of HUVECs. No difference for both Th1 and Tregs was found among migration under the different conditions already tested above in TEM, except for a slight significant pro-migratory effect observed for Th1 in the presence of CAFs_Control_CM compared to the serum-free condition (Figure S2). To understand whether the CAFs secretome can change the expression of surface adhesion molecules of HUVECs that mediate adhesion to and subsequent migration of T cells through HUVECs, we analyzed the mRNA level of E-selectin, ICAM1, and VCAM1 in HUVECs following 2 h of exposure to serum-free medium or CM from CAFs untreated or treated with TGFβ1 and/or LY2157299. No relevant change in expression of these glycoproteins was found according to the strong induction obtained upon stimulation by TNFα and/or IL-1β, used as positive controls (Figure S3).

### 2.3. TGFβ and FoxP3 mRNA Expression Levels Were Inversely Correlated in HCC Tissues

**Figure 3:**
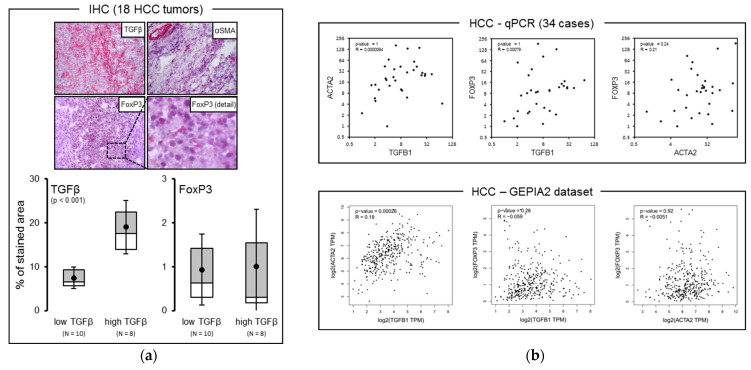

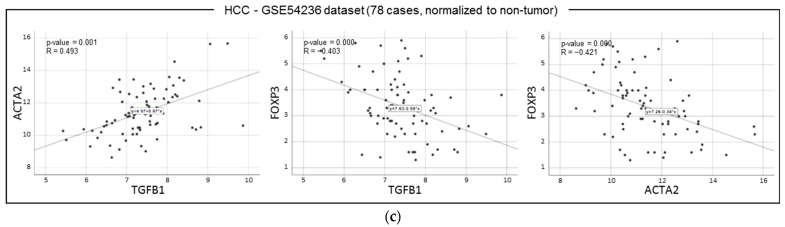
(**a**) TGFβ expression was not associated with FoxP3 expression in HCC tumor tissues. HCC tumor tissues from 18 patients were used. (**b**, upper) TGFβ, αSMA (ACTA2), and FoxP3 mRNA expression levels were not correlated in tumor samples from 34 HCC patients. GAPDH was used as housekeeping gene. (**b**, lower) TGFβ and αSMA expression was positively correlated in HCC tumor samples upon analysis of data available in public dataset GEPIA2 (http://gepia2.cancer-pku.cn/#correlation, accessed on 1 March 2021). (**c**) Analysis of correlation among the same 3 markers in 78 HCC tumor and paired peritumor specimens regarding the dataset GSE54236. In this latter analysis, peritumor mRNA expression values of each marker were subtracted from the matched tumor values for any patient to obtain net tumor expression indexes, as shown in the plots. This setting confirms a positive correlation between TGFβ1 and αSMA mRNA expression level and reveals an inverse correlation between TGFβ1 or αSMA and FoxP3.

We next tried to corroborate the conclusion drawn in in vitro data by visualizing the HCC tissue expression of TGFβ and Tregs (through using FoxP3 staining), as well as retrospectively analyzing HCC tissue mRNA expression levels of TGF-β, aSMA, and FoxP3 in publicly available microarray data (Figure 3). Eighteen HCC tumor tissues were stained for TGFβ, αSMA, and FoxP3 (Figure 3a). No significant correlation was found between FoxP3 and TGFβ expression after stratification of patients according to high and low TGFβ expression (above and under the average expression). Moreover, no significant correlation between TGFβ, αSMA, and FoxP3 mRNA expression was found in a cohort of 34 HCC tumor samples when using GAPDH as housekeeping gene (Figure 3b, upper). Correlation analysis of mRNA expression of the same genes performed via accessing public available GEPIA2 dataset revealed a statistically significant positive correlation limited to TGFβ1 and αSMA (ACTA2) (R = 0.19, *p* < 0.001) (Figure 3b, lower). When we analyzed the GSE54236 dataset, which includes both tumor and surrounding paired non-tumor tissue for any HCC patient, as well as subtracting peritumor from corresponding tumor expression values, we obtained expression indexes for each marker, which were used for subsequent correlation analysis. A significant positive correlation (*p* = 0.000) was found between TGFβ1 and αSMA expression indexes, whereas a significant negative correlation was found for TGFβ-FoxP3 and αSMA-FoxP3 couples (*p* = 0.000) (Figure 3c). These results suggest that HCC, or more generally cancer myofibroblasts, likely counteract the trafficking of FoxP3 Treg cells into the tumor.

### 2.4. Analysis of Secretome of Long-Term TGFβ-Treated CAFs Revealed Changes Influencing Leukocyte Adhesion and Motility

**Figure 4:**
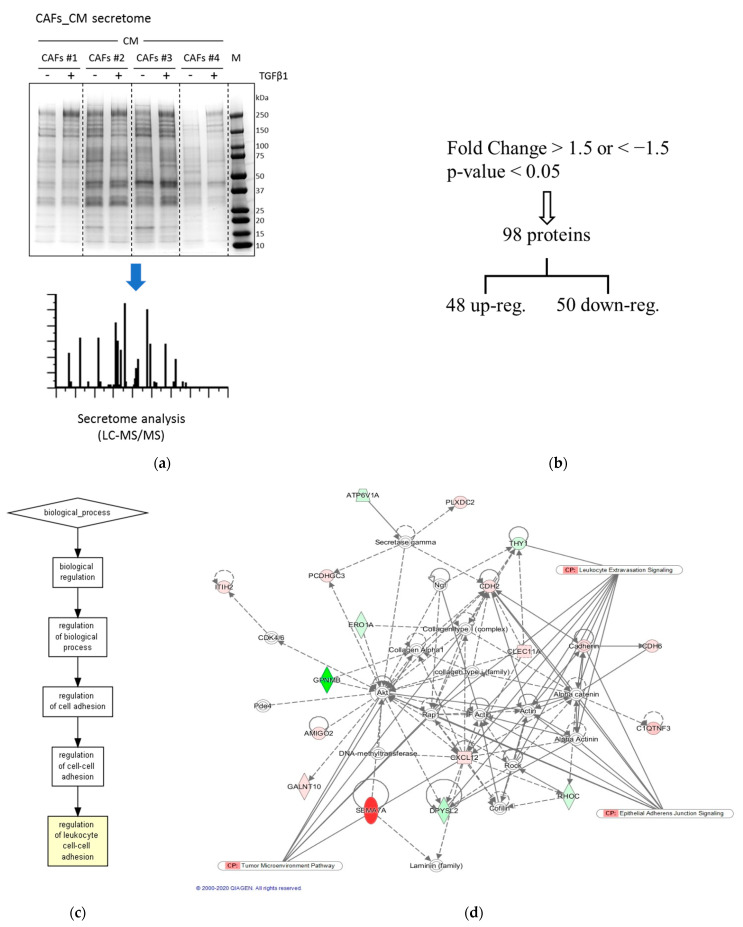
Proteome analysis of CAF secretome upon TGFβ persistent (14 days) stimulation. (**a**) Visualization of secreted proteins from control- and TGFβ1-stimulated CAFs (Coomassie-stained SDS-PAGE gel). (**b**) Output of LC–MS/MS performed on CAF secretome proteins of CAFs persistently (14 days) stimulated with 5 ng/mL TGFβ1. (**c**) Gene Ontology diagram of secretome data processing optimized for leukocyte adhesion. (**d**) Analysis (IPA) of potentially activated pathways in leukocytes as a result of exposure to secretome of HCC CAFs modulated by TGFβ stimulation. TGFβ-modulated interacting signaling hubs are displayed. Up- and downregulated secreted proteins are shown in red and green, respectively.

**Figure 5:**
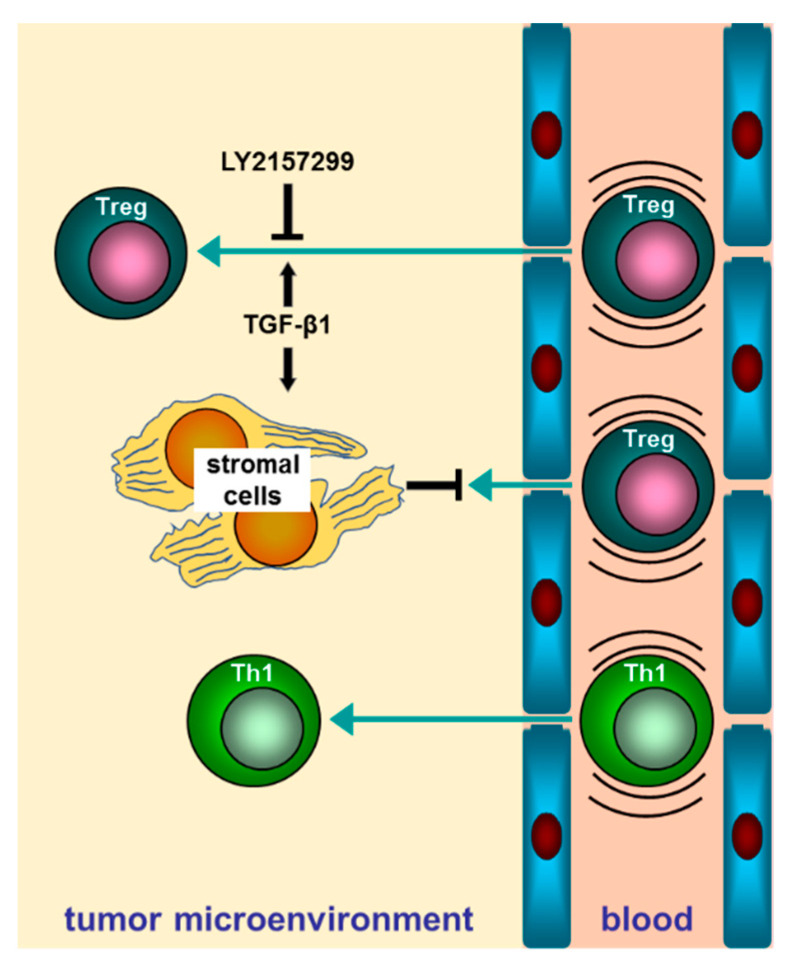
Cartoon depicting direct and stroma-mediated indirect effects of TGFβ on transendothelial motility of Th1- and Treg-orientated CD4 T cells.

Because CAFs_CM_TGFβ1 inhibits the TEM of Tregs compared to CAFs_CM_Ctrl, and the motility of these cells is not ramped up by CAFs_CM_Ctrl, regarding serum-free medium, factors specifically released by TGFβ1-treated CAFs may be responsible for the observed inhibitory effect of CAFs_CM_TGFβ1. Therefore, we hypothesized that factor/s released by CAFs in response to TGFβ stimulus might be responsible for the observed inhibitory effects on migratory potential of Tregs. A whole proteome analysis was performed on proteins secreted by CAFs untreated or previously maintained in a TGFβ-enriched medium for 14 days using a mass spectrometry procedure (LC–MS/MS) (Figure 4). The secretomes of four prior TGFβ-treated or -untreated CAFs deriving from as many corresponding HCC patients were first run in SDS-PAGE and visualized by Coomassie staining (Figure 4a). After LC–MS/MS analysis, we identified 98 differentially expressed secreted proteins that discriminate TGFβ-treated and -untreated CAFs. Specifically, we found 48 significantly upregulated and 50 significantly downregulated proteins by TGFβ (fold change >1.5 or <1.5; *p* < 0.05; Figure 4b, Table S1). In order to identify the molecular mechanisms to which these proteins were involved, we performed a Gene Ontology (GO) analysis and an Ingenuity pathway analysis (IPA, Qiagen). The GO analysis revealed that the biological processes in which the regulated proteins were involved in were the regulation of leukocyte cell–cell adhesion (Figure 4c). Through analyzing the biological functions related to the TGFβ-regulated secretome pattern, we found leukocyte extravasation signaling to be involved, thus suggesting a role of TGFβ-regulated secreted proteins in governing the motility of lymphocytes (Figure 4d). Figure 5 illustrates the direct and indirect (CAF-mediated) dual action of TGFβ on Treg cells transendothelial motility.

## 3. Discussion

TGFβ is long acknowledged as a master regulator of an interactive network involving transformed hepatocytes and multiple other cells populating the HCC microenvironment. Due to the number and complexity of processes affected by this cytokine, its net impact on the malignant evolution of HCC is still not fully elucidated [6]. Tregs abundance in solid tumors is often related to a more suppressed anti-tumor branch of on intra-tumor immunity. TGFβ is required for induction of Tregs and is secreted by these cells when performing suppressive actions [23,24]. Yet, additional properties of Tregs are likely to be influenced by TGFβ. Our data suggest that TGFβ can affect the TEM of Treg-oriented CD4 T cells via at least two counteracting mechanisms: (1) a direct activation of TGFβ receptor, which is required for successful TEM of Treg cells, and (2) a qualitative change of CAF secretome, which in turn impairs Treg migration. The reverse correlation observed in HCC tissues between expression of αSMA and FoxP3 suggests that there may be an increased tendency by more desmoplastic tumors to oppose the homing of Tregs. Consistently, Özdemir et al. have demonstrated that the depletion of carcinoma-associated fibroblasts in mice favors the recruitment of CD4+ FoxP3+ Tregs within experimentally generated tumors [25]. Although the TGFβ is highly expressed in HCC tissues, its activation status in the tumor is not known. This could partially explain the differences in the occurrence of αSMA+ myofibroblasts among the HCCs.

The data here presented reveal a further layer of functional complexity of TGFβ’s role in the context of HCC microenvironment due to its apparently dual counteractive influence on the motility of Tregs. This is antithetical to the evidence that intra-tumor TGFβ is supposed to imprint a tolerogenic phenotype in antigen-presenting cells (mainly dendritic cells), which in turn migrate in peripheral lymphoid tissues to drive FoxP3+ Tregs commitment [26]. The subsequent homing of these cells within the tumor might be the result counterbalancing direct pro-motile, as well as stromal mediated anti-migratory effects of TGFβ. Ongoing clinical trials aim to block TGFβ pathway in solid tumors, including HCC [27,28]. The impact of intratumor Tregs on HCC progression has been widely reported. These cells can inhibit anti-tumor arm of immunity in several ways, mediated by both direct and indirect interactions with multiple leukocyte subsets. A direct contact-mediated inhibition of CD4 Th1 or CD8 cytotoxic T cells (CTLs) by Tregs was described. Membrane-bound TGFβ on the surface of Tregs was reported to exert immunosuppression of some target cells, including natural killer and CD8+ cells [29,30]. This mechanism is likely to occur also into solid tumors. Indirect mechanisms the Tregs exploit to inhibit target cells include: (1) the production of inhibitory cytokines (such as IL-10, IL-35, TGFβ); (2) the sequestration of IL-2, which renders it no longer available for proliferation/maintenance of other immune cells; (3) the binding of CD80/CD86 via CTLA4 on dendritic cells, thus disabling them from activating new effector cells; and (4) the cytolysis of effector cells via secreting granzyme A or B [31].

Although small molecules or antibodies targeting TGFβR have the potential to impair pro-tumor immunity by interfering with the differentiation and functions of Tregs, they do not selectively discriminate between specific cell lineages, thus resulting in indiscriminate shutdown of TGFβ signaling. As this cytokine does not act as an outright tumor suppressor but is rather a dual player depending on the cell type or microenvironmental context, any pharmacological interventions aimed to inhibit its signaling may result in unexpected counterproductive effects. Indeed, a certain activation status of TGFβ signaling in cancer stem cells is reported to refrain their susceptibility to develop chemoresistance and unleash tumorigenic potential [32,33,34,35]. We found that direct inhibition of TGFβ pathway in Treg-oriented CD4 T cells using LY2157299, which specifically blocks the activation TGFβRI chain, reduces TEM of these cells, even in the absence of exogenously added TGFβ. This evidence suggests that Tregs can secrete and respond to TGFβ in an autocrine manner. As the TGFβ receptor complex activation takes place following the assembly of TGFβ/TGFβRI/TGFβRII, and the TGFβRII receptor chain is not targeted by LY2157299 and can signal regardless of TGFβRI, it is clear that the reduced motility observed for Treg is, at least partially, dependent on TGFβRI-associated signaling.

After normalization of mRNA expression values of TGFβ1 and FoxP3 of HCC tumors to the values of matched peritumor tissues, we found an inverse correlation between the net tumor expression of these markers (Figure 3c). This is in apparent contrast with the findings of previous studies. The authors of these reports found that TGFβ1 and FoxP3 absolute protein expression levels are positively correlated in HCC tissues, thus suggesting TGFβ involvement in Treg accumulation [22,36]. Our data, on the other hand, consider the potential contribution of TGFβ of tumor origin only, subtracted of the peritumor background, on the tissue enrichment of FoxP3+ cells. As a possible explanation for the above discrepancy, we hypothesize that the secretome of CAFs reprogrammed by the hyperactivation of the TGFβ pathway occurring within the tumor may counteract the accumulation of intratumor Tregs in vivo, likely prevailing over the direct action of TGFβ in attracting these cells. The perspective of conceiving more effective immuno-targeting therapies in HCC treatment might benefit from a stratification of patients based on both the presence and functional status on intra-tumor immune cells subsets and the expression of micro-environmental factors such as TGFβ that impact on the activity of these cells.

## 4. Materials and Methods

### 4.1. List of Antibodies and Other Reagents

Antibodies: mouse anti-vimentin, mouse anti-cytokeratin, mouse anti-CD45, mouse anti-alpha smooth muscle actin (αSMA); rabbit anti-CD11b, rabbit anti-N-cadherin (Abcam, Cambridge, MA, USA); mouse anti-FoxP3-AF647, mouse IgG-AF647 isotype antibodies (BD Biosciences, San Diego, CA, USA); rabbit anti-T-bet (Cell Signaling, Danvers, MA, USA); chicken anti-mouse AF-488-conjugated, goat anti-rabbit AF-594-conjugated (Thermo Fisher Scientific, Waltham, MA, USA). Cytokines: human recombinant interleukin-2 and -12 (IL-2, IL-12), transforming growth factor beta-1 (TGFβ1) (Peprotech EC, London, UK). Drugs: LY2157299 (galunisertib, Cayman Chemical Company, Ann Arbor, MI, USA).

### 4.2. CAFs Isolation

Human HCC tissue specimens were collected from primary tumors at time of surgical resection and were minced in Hanks’ balanced salt solution (HBSS) to obtain <5 mm size pieces. These samples were washed twice in HBSS and then incubated for 2 h at 37 °C in gentle rotation in HBSS in the presence of collagenase IV (Life Technologies, Eugene, OR, USA), 3 mM CaCl_2_, and antibiotic-antimycotic agent (Euroclone, Milan, Italy). Cells released from dissociation were washed with complete IMDM (+20% FBS), re-suspended in the same medium, and temporally kept on ice (first round of digestion). Partially digested tissues were subjected to a second 2 h digestion round. The cells released were then washed with complete IMDM medium, combined with the cells obtained from the first digestion step, and incubated in normal culture condition. A few passages (2–3) were sufficient for eventually adhered epithelial cells (tumor cells, hepatocytes) to undergo apoptosis and detach. The resulting CAF-enriched population was characterized for expression of mesenchymal markers (vimentin, αSMA), and the presence of minimal contaminating non-fibroblastic cells (such as cholangiocytes and macrophages) was assessed by immunofluorescence and flow cytometry (using antibodies to CD45, CD11b, and cytokeratin) (Figure 1). 

CAF treatment and CM obtainment. CAFs were treated for 14 days in complete IMDM medium, in the presence of DMSO, or 10 µM LY2157299 (galunuisertib, Lilly), or 5 ng/mL TGFβ1 (+ DMSO), or both TGFβ1 + LY2157299. Medium and treatments were renewed every 2 days. At the end of treatment, CAFs were washed four times with PBS left in serum-free IMDM to obtain conditioned medium (CM). CM was concentrated by using 15 mL 10 kDa cutoff centricon (Merck-Millipore), then assayed for protein concentration.

### 4.3. Obtainment of CD4 Th1 or Treg-Oriented Cells

Peripheral blood mononuclear cells (PBMCs) were obtained from freshly withdrawn blood of normal subject by using Lympholyte (Cedarlane, Burlington, ON, Canada) according to the user’s guide. CD4+ T cells were then purified from PBMCs by using *Dynabeads Untouched Human CD4 T Cells* (Life Technologies). CD4+ T cells were then incubated for 7–9 days with *Dynabeads Human T-Activator CD3/CD28* (Life Technologies) in the presence of 5 µM IL-12 + 5 µM IL-2 to generate Th1-oriented phenotype, or 5 µM IL-12 + 5 µM TGFβ1 to generate Treg-oriented phenotype (Figure 1a). Cells were cultured in RPMI medium.

### 4.4. Transendothelial Migration Assay

Human umbilical vein endothelial cells (EndoGRO HUVECs, cultured in EndoGRO medium, Merck KGaA, Darmstadt, Germany) were seeded on the upper side of porous (8 µm pores size) polycarbonate membranes (10^5^ cells per membrane) of 24-well suitable culture inserts (transwells, Corning Incorporated, Corning, NY, USA) and incubated for 24 h to form a tight layer. Four hundred microliters of RPMI medium (+0.5% bovine serum albumin) with/without 30 µg/mL of total CAF secreted proteins (from CAF conditional medium) or 5 ng/mL TGFβ1 and/or 10 µM LY2157299 (galunisertib) was added in the lower chamber. The HUVEC medium was then removed, and a volume of 200 µL of lymphocyte suspension (10^5^ cells) was loaded onto the HUVECs layer. After 16 h, the trans-migrated lymphocytes were collected from the lower chamber, then spun down, re-suspended in a small medium volume (<100 µL), and counted. The total number of migrated cells was determined according to the volume.

### 4.5. RNA Extraction and Real-Time Quantitative PCR (qPCR)

RNA was extracted from HCC cells and tissues using RNeasy mini kit (Qiagen, Venlo, The Netherlands) according to manufacturer’s instructions. Two micrograms of RNA were retrotranscribed for cDNA synthesis using the High-Capacity cDNA Reverse Transcription Kit (Thermo Fisher Scientific, USA). Real-time qPCR was carried out in 20 µL final volume using 1 ng/µL cDNA and forward + reverse primers (500 nM each) using iTaq Universal SYBR Green Supermix (Bio-Rad, Laboratories (Hercules, CA, USA).List of sequences of used primers (5′–3′): GAPDH (For)—CACCATCTTCCAGGAGCGAG; GAPDH (Rev)—GACTCCACGACGTACTCAGC; E-Sel (For)—AGCTCTCACTTTGGTGCTTCT; E-Sel (Rev)—TAGCTTCCGTGGAGGTGTTG; ICAM-1 (For)—AGCTTCGTGTCCTGTATGGC; ICAM-1 (Rev)—TTTCTGGCCACGTCCAGTTT; VCAM-1 (For)—CGAACCCAAACAAAGGCAGAG; VCAM-1 (Rev)—CTCTGGGGGCAACATTGACA; GATA3 (For)—GGCAGGGAGTGTGTGAACTG; GATA3 (Rev)—GCCTTCGCTTGGGCTTAATG; T-bet (For)—GAGGTGTCGGGGAAACTGAG; T-bet (Rev)—ATGGGAACATCCGCCGTC; FoxP3 (For)—AACGGTGGATGCCCACG; FoxP3 (Rev)—GGCCACGTTGATCCCAGG; CD25 (For)—GCAGTGGCAACCTTGTCTCTATG; CD25 (Rev)—GGTTTTGCCCTTCCTCTTCAAC; TGFβ1 (For)—GGAAATTGAGGGCTTTCGCC; TGFβ1 (Rev)—GGTAGTGAACCCGTTGATGTCC.

### 4.6. Flow Cytometry Analysis

CAFs were fixed for 15 min with 4% paraformaldehyde in PBS, pH = 7.4, then permeabilized/blocked for 20 min with 0.1% Triton-X100 + 2% BSA in PBS (perm/block buffer), then incubated with primary anti-αSMA antibody and subsequently with secondary fluorophore (AF488)-conjugated antibody, or with only secondary antibody (as control sample) diluted in perm/block buffer. Lymphocytes were fixed/permeabilized with Foxp3/Transcription Factor Staining Buffer Set (eBioscience, Thermo Fisher Scientific, USA), and then stained using primary fluorophore-conjugated antibodies (anti-FoxP3 and its related isotype control antibody), or unconjugated primary antibodies prior to incubation with secondary conjugated antibodies (anti-T-Bet and anti-N-chadherin as related isotype control antibody). Cells were analyzed using Accuri C6 cytometer (BD, USA) or Navios flow cytometer (Beckman Coulter). Accuri, FlowJo (BD, USA), and Kaluza (Beckman Coulter Inc., Brea, CA, USA) software were employed for the quantitative analysis.

### 4.7. LC–MS/MS Mass Spectrometry

Proteins in concentrated CAFs_CMs were trypsin digested and analyzed with 0.5 h LC–MS/MS. These steps were performed by MSBioworks (3950 Varsity Dr, Ann Arbor, MI 48108). Cellular signaling pathways of interest were analyzed using *Ingenuity Pathway Analysis (IPA)* software (Qiagen, Hilden, Germany).

### 4.8. Statistical Analysis

*t*-test (two tails, paired) was used for analysis of in vitro cell culture experiments. Pearson correlation (two-tailed) was used to measure the linear correlation coefficient between couples of mRNA expression datasets referred to human tumor and matched peritumor liver tissues.

### 4.9. Ethics Approval

This study was approved by the local ethics committee, Azienda Ospedaliero Universitaria Consorziale Policlinico di Bari (Bari, Italy); protocol number: 254; date of release: February 2012.

## Data Availability

The mass spectrometry proteomics data have been deposited to the ProteomeXchange Consortium via the PRIDE [37] partner repository with the dataset identifier PXD029586.

## Supplementary Materials

The following are available online at www.mdpi.com/article/10.3390/ijms222111765/s1.
