# Supplementary material for: Direct and Indirect Effect of TGFβ on Treg Transendothelial Recruitment in HCC Tissue Microenvironment
Source: Int J Mol Sci. 2021 Oct 29;22(21):11765. doi: 10.3390/ijms222111765 (PMC8583957; doi:10.3390/ijms222111765)
Supplement: Supplementary file 1 [file ijms-22-11765-s001.zip › ijms-1404469-Supplementary File.pdf]

Supplementary Figures/Tables

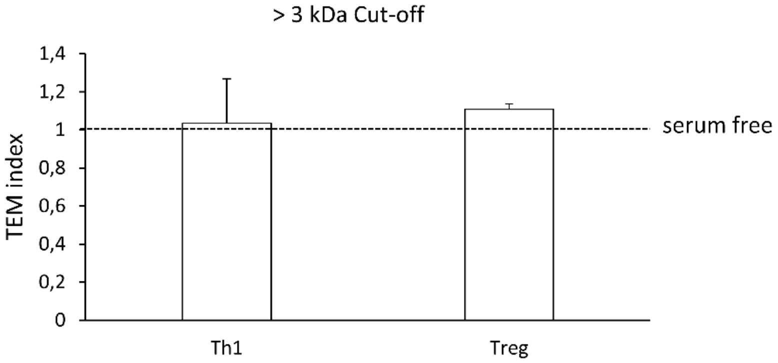

Supplementary Figure S1.

CAFs-conditioned medium retaining proteins with a molecular weight > 3 kDa used as a chemoattractant does not significantly affect the transendothelial migration of Th1 and Treg-oriented CD4T cells compared to basal medium.

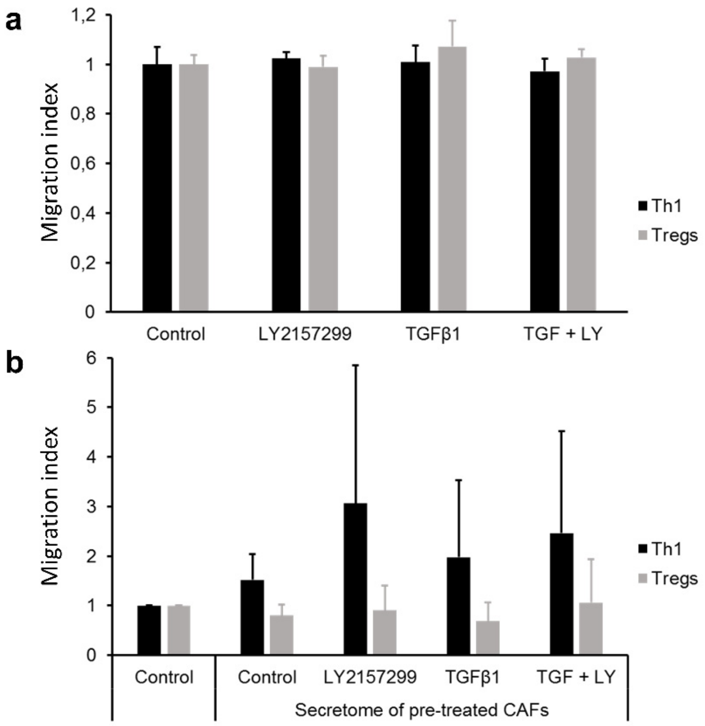

Supplementary Figure S2.

Endothelial cells mediate the effects of TGFβ and conditioned medium of TGFβ-educated CAFs on transendothelial migration of Treg cells. Migration assays were performed using transwells with 3 μm diameter pores. (a) Exogenously added TGFβ1 and/or galunisertib (LY2157299) do not significantly affect migration of Th1 and Treg oriented CD4 T cells in

the absence of HUVECs endothelial layer. Data are expressed as means (normalized on the control) + SD from triplicates. (b) Conditioned medium of TGFβ1 and/or galunisertib pre-treated CAFs does not significantly affect migration of Th1 and Treg oriented CD4 T cells in the absence of HUVECs endothelial layer. Data are expressed as means (normalized on the control) + SD of 6 independent assays performed using conditioned medium from CAFs obtained from as many HCC patients.

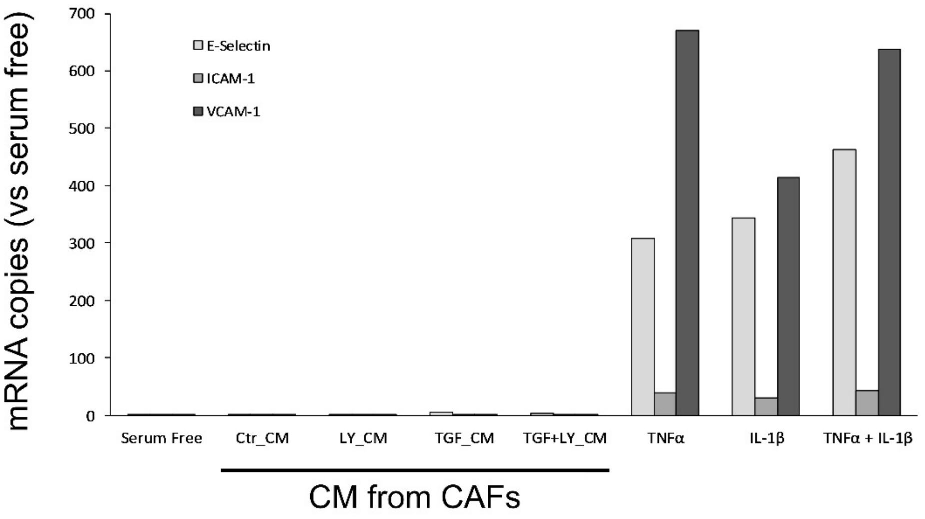

Supplementary Figure S3.

Conditioned medium of TGFβ1/galunisertib pre-treated CAFs does not induce the expression of adhesion receptors for leukocytes (E-selectin, ICAM-1 and VCAM-1) on endothelial cells.

Supplementary Table S1.

List of proteins down- and up-regulated in conditioned medium of CAFs from 4 HCC patients in response to long term TGFβ1 stimulation. The amount of proteins secreted by untreated cells was used as reference (control). A threshold of ±1.5 in fold change versus control and only statistically significant changes ($p < 0.05$) were selected to consider variations in protein abundance of interest.

Down-regulated proteins

Accession Number	Gene ID	p-value	Fold Change
Q14956	GPNMB	0,0186	-21,6
Q14108	SCRB2	0,0460	-19,5
P62701	RS4X	0,0374	-13,2
Q9NZM1	MYOF	0,0338	-10,0
P21796	VDAC1	0,0460	-8,54
Q9BT78	CSN4	0,0091	-8,29
Q96QK1	VPS35	0,0401	-8,25
P63092	GNAS2	0,0080	-7,11
Q7Z4H3	HDDC2	0,0358	-6,97
O95297	MPZL1	0,0343	-5,70
O95831	AIFM1	0,0406	-5,21
Q16555	DPYL2	0,0284	-5,01
P61978	HNRPK	0,0004	-5,00
Q9NR28	DBLOH	0,0096	-4,81
Q9BQ51	PD1L2	0,0448	-4,56
P00750	TPA	0,0025	-4,54
P07741	APT	0,0491	-4,31
P13674	P4HA1	0,0020	-4,09
O60613	SEP15	0,0374	-3,97
O15460	P4HA2	0,0009	-3,70
Q9BXJ1	C1QT1	0,0081	-3,69
Q7Z5L7	PODN	0,0268	-3,52
P38606	VATA	0,0320	-3,44
P62913	RL11	0,0300	-3,39
Q14112	NID2	0,0422	-3,33
P05362	ICAM1	0,0494	-3,26
P04179	SODM	0,0001	-3,13
P07437	TBB5	0,0198	-2,86
Q9BS40	LXN	0,0144	-2,85
Q96HE7	ERO1A	0,0364	-2,82
P15018	LIF	0,0166	-2,78
P46926	GNPI1	0,0047	-2,65
P19367	HXK1	0,0265	-2,24
P02792	FRIL	0,0465	-2,23
P04216	THY1	0,0306	-2,21
P10301	RRAS	0,0008	-2,19
P28062	PSB8	0,0128	-2,19
P08134	RHOC	0,0401	-2,17
Q14847	LASP1	0,0353	-2,15
P05231	IL6	0,0239	-2,10
P14618	KPYM	0,0232	-2,06
Q9Y4K0	LOXL2	0,0095	-2,04
P30740	ILEU	0,0266	-2,02
Q13162	PRDX4	0,0412	-2,01
Q13813	SPTN1	0,0269	-1,74
P30626	SORCN	0,0021	-1,69
P14625	ENPL	0,0209	-1,67
Q15293	RCN1	0,0451	-1,66

Up-regulated proteins

Accession Number	Gene ID	p-value	Fold Change
O75326	SEM7A	0,0000	75,7
Q9BXJ4	C1QT3	0,0110	20,8
Q86Y38	XYLT1	0,0003	15,8
Q8N6Y2	LRC17	0,0423	11,5
O75063	XYLK	0,0040	9,15
Q8IUX7	AEBP1	0,0257	8,46
Q9UNZ2	NSF1C	0,0014	7,36
Q9UN70	PCDGK	0,0380	5,55
P49746	TSP3	0,0194	5,02
P19823	ITIH2	0,0062	4,93
Q16769	QPCT	0,0102	4,49
Q9UQ80	PA2G4	0,0142	4,47
Q9H013	ADA19	0,0058	4,45
P55285	CADH6	0,0016	4,40
O14498	ISLR	0,0104	4,35
P01137	TGFB1	0,0009	4,17
P00747	PLMN	0,0211	4,13
P00488	F13A	0,0329	4,13
Q86SR1	GLT10	0,0084	3,97
P54687	BCAT1	0,0014	3,83
P21810	PGS1	0,0000	3,55
Q14767	LTBP2	0,0017	3,13
Q9NS15	LTBP3	0,0355	3,10
Q15113	PCOC1	0,0210	3,09
Q13438	OS9	0,0318	3,04
Q9NRN5	OLFL3	0,0141	2,97
P48061	SDF1	0,0305	2,86
Q86SJ2	AMGO2	0,0317	2,84
Q8N6T3	ARFG1	0,0394	2,69
Q53FA7	QORX	0,0139	2,65
Q01469	FABP5	0,0130	2,48
Q15262	PTPRK	0,0153	2,45
P43034	LIS1	0,0153	2,43
Q6UX71	PXDC2	0,0357	2,40
O43184	ADA12	0,0351	2,30
P31153	METK2	0,0292	2,30
O75874	IDHC	0,0061	2,26
P07910	HNRPC	0,0490	2,25
Q6IBS0	TWF2	0,0021	2,15
P20908	CO5A1	0,0007	2,06
P19022	CADH2	0,0448	1,96
P02751	FINC	0,0103	1,94
P43251	BTD	0,0362	1,86
P13497	BMP1	0,0391	1,83
P06396	GELS	0,0293	1,82
P08476	INHBA	0,0404	1,81
P54819	KAD2	0,0437	1,70
P07093	GDN	0,0193	1,68
Q9Y240	CLC11	0,0450	1,61
Q08629	TICN1	0,0075	1,55
